# Analysis of *LOXL1* polymorphisms in a Saudi Arabian population with pseudoexfoliation glaucoma

**Published:** 2010-12-16

**Authors:** Khaled K. Abu-Amero, Essam A. Osman, Ahmed S. Dewedar, Silke Schmidt, R. Rand Allingham, Saleh A. Al-Obeidan

**Affiliations:** 1 Ophthalmic Genetics Laboratory, College of Medicine, King Saud University, Riyadh, Saudi Arabia; 2 Glaucoma Unit, Department of Ophthalmology, College of Medicine, King Saud University, Riyadh, Saudi Arabia; 3 Center for Human Genetics, Duke University Medical Center, Durham, NC; 4 Department of Ophthalmology, Duke University Eye Center, Durham, NC

## Abstract

**Purpose:**

To investigate whether single nucleotide polymorphisms (SNPs) in the lysyl oxidase-like 1 (*LOXL1*) gene are associated with pseudoexfoliation glaucoma (PEG) in the Saudi Arabian population.

**Methods:**

The coding regions of *LOXL1* were fully sequenced in 93 clinically diagnosed PEG patients and 101 healthy controls. Both groups were Saudi Arabs. Previously reported and newly identified SNPs were evaluated for possible association with PEG and their pathological consequences on the gene were assessed.

**Results:**

The “G” allele frequencies of both rs1048661 and rs3825942 SNPs differed between PEG patients and control subjects from Saudi Arabia (p=0.0056 and p=0.000005, respectively). This significance remained after applying the Bonferroni correction. Two non-synonymous novel SNPs in *LOXL1* were detected in the PEG patients and not in the controls. One of these SNPs was in exon 4 (g.25722 C>G; codon change D484E) of *LOXL1* and was predicted to be non-pathological; the other was in exon 6 of *LOXL1* (g.28084 T>G; codon change Y559D) and was predicted to be probably damaging. All alleles of SNPs (rs28706550, rs35203737, rs41429348, rs12906373, rs41435250, and rs13329473) were monoallelic in this population. No allele frequency difference for rs8818 and rs3522 SNP between patients and controls (p values were 0.126 and 0.994 respectively).

**Conclusions:**

Similar to almost all non-African populations tested thus far, the “G” allele of both rs1048661 and rs3825942 SNPs were associated with the risk of PEG in the Saudi Arab population.

## Introduction

Pseudoexfoliation syndrome (XFS) is characterized by deposits of grayish-white material seen primarily in the anterior segment of the eye. The deposits are primarily found along the pupillary border and often produce a characteristic pattern on the anterior lens surface [1]. XFS is frequently associated with pseudoexfoliation glaucoma (PEG), which often has a more aggressive clinical course and worse prognosis than the more common primary open angle glaucoma (POAG) [2]. The incidence of PEG in XFS patients varies and seems highest among individuals with Scandinavian and Northern European ancestry and lower among African Americans and in West Africa [3-5]. The prevalence of PEG in Saudi Arabia is unknown. The Glaucoma unit at King Abdulaziz University Hospital (where most PEG patients were recruited for this study) sees around 600 new glaucoma patients every year, and about 10% of those are PEG.

Thorleifsson and colleagues [6] have reported a genome-wide association study that identified a strong association between three single nucleotide polymorphisms (SNPs) in the lysyl oxidase-like 1 (*LOXL1)* gene. They identified one intronic SNP (rs2165241) and two non-synonyrnous coding SNPs (rs1048661 and rs3825942) with significant disease association in Icelandic and Swedish subjects. *LOXL1* belongs to the “LOX” family of extracellular enzymes that have multiple functions including the cross-linking of collagen and elastin by oxidatively deaminating lysine residues. Since XFS deposits are associated with the extracellular and basement membrane regions, the LOX genes are legitimate functional candidates to be involved with PEG pathogenesis [7].

The association of *LOXL1* SNPs (in particular rs1048661 and rs3825942) with XFS/PEG has now been studied in Caucasian populations in the USA [8], Australia [9], Austria [10], Germany [11], Italy [12], and Finland and in other ethnic groups, including Japanese [13], Indian [14], Chinese [15], and recently black South Africans [16]. The “G” allele of SNP rs3825942 is significantly associated with XFS/PEG in all populations tested to date [17] with the exception of black South Africans where the “A” allele is the risk allele [16].

The “G” allele of SNP rs1048661 is associated with XFS/PEG in all populations except in the Indian [14] and Chinese [15] populations. In other studies, the opposite “T” allele of SNP rs1048661 was shown to be the risk allele for PEG in the Chinese [18] and Japanese [19] populations.

This study was conducted to examine the frequency of various polymorphisms of *LOXL1* in the Saudi Arabian population with PEG and to evaluate whether SNPs in the *LOXL1* gene associated with the risk of PEG in this population.

## Methods

### Study population

The study adheres to the tenets of the Declaration of Helsinki, and all participants signed an informed consent. The study was approved by College of Medicine ethical committee (proposal number # 08–657). All study subjects were self identified as Saudi Arabian ethnicity. Family names were all present in the database of Arab families of Saudi Arabian origin. Additionally, these names indicated that all five major Saudi Arabian provinces were represented in the study population. Expatriates were excluded from this study and all patients and controls were Saudi Arabs. Subjects with clinically diagnosed PEG and healthy controls were recruited into the study at King Abdulaziz University Hospital in Riyadh, Saudi Arabia. All participants underwent a standardized detailed ophthalmic examination, which included measurement of intraocular pressure (IOP) by Goldmann applanation tonometry, slit lamp biomicroscopy, gonioscopy, and dilated examination of the lens and fundus. Subjects with PEG were defined as those with clinical evidence of exfoliation material on the pupil margin or anterior lens surface and the presence of glaucomatous optic neuropathy with associated visual field loss in one or both eyes and documented IOP ≥22 mmHg in either eye. Saudi Arab subjects with normal anterior segment and optic nerve examination, IOP <18 mmHg, and no clinical signs of exfoliation were recruited as control subjects.

### DNA analysis

Five ml of peripheral blood were collected in EDTA tubes from all participating individuals. DNA was extracted using the illustra blood genomicPrep Mini Spin Kit from GE Healthcare (Buckinhamshire, UK), and stored at −20 °C in aliquots until required. PCR amplifications of the 7 exons and the promoter region of *LOXL1* were performed using the primers listed in Table 1. Successfully amplified fragments were sequenced in both directions using the M13 forward and reverse primers and the BigDye terminator v3.1 cycle sequencing kit (Applied Biosystems, Foster city, CA). Fragments were then run on the 3130xl Genetic Analyzer (Applied Biosystems) according to the manufacturer protocol. All the sequenced fragments were then analyzed using SeqScape software v2.6 (Applied Biosystems). Allele frequencies for SNP rs1048661 and rs3825942 were confirmed by repeating the sequencing in both the forward and reverse directions. Table 1 details the sequence of the primers used, the PCR annealing temperature, and the expected amplicon size.

**Table 1:** Primer sequences, PCR annealing temperature, and amplicon size for *LOXL1*.

**Exon**	**Primer sequence**	**Annealing temperature (°C)**	**Amplicon size (bp)**
Promoter-F	**TGTAAAACGACGGCCAGT**AGGGACTGAGGGAGCACT	60	465
Promoter-R	**CAGGAAACAGCTATGACC**AGCCATGGTGACCCCTCT		
1A-F	**TGTAAAACGACGGCCAGT**TCCCAGCCTGTTGCTTATTC	60	789
1A-R	**CAGGAAACAGCTATGACC**GTTGCTGGGAGACGGAGGT		
1B-F	**TGTAAAACGACGGCCAGT**ATTCGGCTTTGGCCAGGT	60	810
1B-R	**CAGGAAACAGCTATGACC**CCGGAGCAGTTTCCAGTG		
1C-F•	**TGTAAAACGACGGCCAGT**GCTCAACTCGGGCTCAGA	57	553
1C-R•	**CAGGAAACAGCTATGACC**CGCCGTACTCCTCGTAGC		
2-F	**TGTAAAACGACGGCCAGT**TGCTCTCAATGTCATGCTCTT	60	209
2-R	**CAGGAAACAGCTATGACC**CGGGGACTATCCCAACACT		
3-F	**TGTAAAACGACGGCCAGT**GTGTCACTGTGCCCCAACC	60	232
3-R	**CAGGAAACAGCTATGACC**CCCAGAGGAGAAGTGGAAGA		
4-F	**TGTAAAACGACGGCCAGT**GAGAGGCCCAGGGAAGACTA	58	265
4-R	**CAGGAAACAGCTATGACC**CCTCCCCAACTCCTTATCCT		
5-F	**TGTAAAACGACGGCCAGT**GGGGTGGCTCTGGGAAAC	58	210
5-R	**CAGGAAACAGCTATGACC**GGGGGACATTGGACATGA		
6-F	**TGTAAAACGACGGCCAGT**CCCCTGACTAGACTCCCTTTC	60	234
6-R	**CAGGAAACAGCTATGACC**GTATCTCAGGTGGCCTTGC		
7-F	**TGTAAAACGACGGCCAGT**CTACTTTGCAGCCCCTCATT	60	410
7-R	**CAGGAAACAGCTATGACC**CCAGGCCCAAACTAGCTG		

In the table, F: Forward; R: Reverse; ^•^SNPs rs1048661 and rs3825942 were amplified with this primer set. Bold and underlined sequences are those of M13

### Statistical analysis

Allele frequencies in study groups were compared using the χ^2^ test. A two tailed p value <0.05 was considered statistically significant. An exact test for Hardy–Weinberg equilibrium (HWE) of the observed genotypes was performed. Given the known high consanguinity rate in Saudi Arabia (>65% in some areas; see Panter [20]) and the possible absence of random mating in this population, we also calculated allelic association p values that were adjusted for deviation from HWE using the method described in Schaid and Jacobsen [21]. All analyses were performed using SPSS v.10 (SPSS Inc., Chicago, IL) and SAS v.9.1 (SAS Institute Incl, Cary, NC) statistical analysis software. Common polymorphisms of *LOXL1* were subjected to Bonferroni correction for multiple testing.

## Results

Ninety three PEG patients and 101 controls were recruited into this study. Of the 93 PEG patients there were 61 males and 32 females with a mean age of 72.3 (SD 12.02). Of the 101 controls there were 64 males and 37 females with a mean age of 69.3 (SD 12.4). The full coding region, exon-intron boundaries, the promoter region (470 bases before the transcriptional start site), and the 5′UTR and 3′UTR of the *LOXL1* gene was sequenced in all subjects.

The “G” allele frequencies of both rs1048661 (0.876) and rs3825942 (0.968) SNPs differed significantly between PEG patients and controls subjects (p=0.0056 and 0.000005, respectively). If we consider the 4 common SNPs (rs1048661, rs3825942, rs28706550, and rs35203737), then the Bonferroni corrected p-value should be 0.0125. After applying the Bonferroni correction, SNPs rs1048661 and rs3825942 remained significant.

There was no statistically significant difference in genotype frequencies between patients and controls for SNP rs1048661 (p values were 0.409 and 1.000 for genotypes G/G and G/T respectively). As for SNP rs3825942, there was a statistically significant difference between patients and controls for the G/G genotype (p=0.049), but that significance disappeared after applying Bonferroni correction. As for the G/A genotype of SNP rs3825942, there was no difference in genotype frequency between patients and controls (p=1.000). Genotypes of SNPs rs28706550 and rs35203737 were monogeneic in all patients and controls (Table 2).

**Table 2:** Genotype frequencies of the four most common LOXL1 SNPs.

**SNP I.D**	**Nucleotide change**	**Genotype**	**XFG patients (n=93)**	**Controls (n=101)**	**p value**
rs1048661	g.5758 G>T	G/G	72 (77.4%)	57 (56.4%)	0.409
		G/T	19 (20.4%)	40 (39.6%)	1.000
		T/T	2 (2.2%)	4 (4%)	reference
rs3825942	g.5794 G>A	G/G	88 (94.6%)	70 (69.3%)	0.049
		G/A	4 (4.3%)	25 (24.7%)	1.000
		A/A	1 (1.1%)	6 (6%)	reference
rs28706550	g.25067 A>C	A/A	93 (100%)	101 (100%)	-
		A/C	0 (0)	0 (0)	-
		C/C	0 (0)	0 (0)	reference
rs35203737	g.28103 C>A	C/C	93 (100%)	101 (100%)	-
		C/A	0 (0)	0 (0)	-
		A/A	0 (0)	0 (0)	reference

Alleles of SNPs rs28706550, rs35203737, rs41429348, rs12906373, rs41435250, and rs13329473 were monoallelic in all PEG subjects and controls.

There was no significant difference in allele frequencies between cases and controls for the rs8818 and rs3522 SNPs (p values were 0.126 and 0.994, respectively; Table 3).

**Table 3:** Allele frequencies of various *LOXL1*-SNPs in PEG patients and controls.

**SNP I.D.**	**Nucleotide change**	**Amino acid change**	**Allele**	**PEG patients (n=93)**	**Controls (n=101)**	**p value**
rs1048661	g.5758 G>T	R141L	G	163 (0.876)^•^	154 (0.762)	0.0056
rs3825942	g.5794 G>A	G153D	G	180 (0.968)	165 (0.817)	0.000005
rs28706550	g.25067 A>C	N437H	A	186 (1)	202 (1)	N/A
rs35203737	g.28103 C>A	S565Y	C	186 (1)	202 (1)	N/A
Novel	g.25722 C>G	D484E	C	184 (0.99)	202 (1)	N/A
Novel	g.28084 T>G	Y559D	T	185 (0.995)	202 (1)	N/A
rs8818	g.30490 C>G	-	G	141 (0.758)	138 (0.683)	0.126
rs3522	g.30556 C>T	-	C	116 (0.624)	125 (0.619)	0.994
rs41429348	g.6212 C>T	-	C	186 (1)	202 (1)	N/A
rs12906373	g.6272 C>T	-	C	186 (1)	202 (1)	N/A
rs41435250	g.6296 G>T	-	G	186 (1)	202 (1)	N/A
rs13329473	g.25737 C>T	-	C	186 (1)	202 (1)	N/A

*LOXL1* indicates lysyl oxidase-like 1; PEG indicates exfoliation glaucoma. Alleles in bold were considered the risk alleles in calculating p value. Nucleotide are numbered as in GenBank accession number NG_011466. ^•^Numbers in parenthesis represent the allele frequency. Novel indicates not previously reported. N/A indicates not applicable.

Genotypes at SNPs rs1048661, rs3825942, rs8818, and rs3522 were in HWE for both patients and controls (p≥0.05). Since the test for deviation from HWE has limited statistical power for moderate sample sizes, and since non-random mating may exist in the study population [20], we also calculated allelic association p-values that are adjusted for deviation from HWE. The results (p=0.00257 for rs1048661 and p=0.00000479 for rs3825942) indicated that the presence of allelic association was robust to the absence or presence of HWE.

We detected two previously unreported SNPs in this population. The rare allele was found in PEG subjects but not the controls for both SNPs. The first SNP is g.25722 C>G in exon 4 of the *LOXL1* gene and results in a replacement of aspartic acid with glutamic acid at codon 484 (D484E). This SNP was detected in 2/93 (2.15%) of PEG patients and was predicted by PolyPhen to be benign with no pathological consequences on the protein structure and/or function (Table 4). The second novel SNP g.28084 T>G was detected in exon 6 of the *LOXL1* gene and resulted in a replacement of tyrosine with aspartic acid at codon 559 (Y559D). This SNP was detected in one PEG patient (1.1%), but not in the controls. Polyphen predicted this SNP to be probably damaging (Table 4) and thus may affect the protein structure and/or function.

**Table 4:** Analysis of non-synonymous sequence changes in the LOXL1 gene detected in the study group.

**SNP ID**	**Nucleotide sequence change**	**Amino acid change**	**Location**	**Interspecies conservation**	**PolyPhen prediction**
rs1048661	g.5758 G>T	R141L	Exon 1	High	Probably damaging
rs3825942	g.5794 G>A	G153D	Exon 1	High	Possibly damaging
Novel	g.25722 C>G	D484E	Exon 4	High	Benign
Novel	g.28084 T>G	Y559D	Exon 6	High	Probably damaging

Non-synonymous indicates sequence change which results in an amino acid change. PolyPhen=Polymorphism Phenotyping is a tool which predicts possible impact of an amino acid substitution on the structure and function of a human protein using straightforward physical and comparative considerations. “Probably damaging” constitutes high confidence of affecting protein function. “Possibly damaging” reflects a likelihood of affecting protein function or structure, while “Benign” changes most likely lack phenotypic effect.

## Discussion

To our knowledge, this is the first study to evaluate whether SNPs in *LOXL1* associated with the risk of PEG in the Saudi Arab population. We first evaluated the prevalence of non-synonymous SNPs in *LOXL1* in PEG patients and glaucoma-free controls and compared the results in both groups. The results, presented in Table 3, indicate that the prevalence of the “G” allele of SNP rs1048661 in PEG patients (0.876) was higher than the controls (0.762) and that this difference was statistically significant (p=0.0056). After applying Bonferroni correction, the genotype frequencies were comparable in patients and controls and the difference was not statistically significant (Table 2). This indicates that the allele and not the genotype may contribute or act as a risk for the disease. The “G” allele frequency of 0.876 observed in Saudi Arabian PEG patients was comparable to the rate observed in Icelandic (0.781), Swedish (0.834), Americans (0.819), Austrians (0.841), Germans (0.818), Italians (0.825), Finnish (0.825), and South Africans (0.990) [17]. However, it was higher than the rate observed in Chinese (0.110) and Japanese (0.036) populations [17]. This indicates that the variability of the “G” allele frequencies depends on region and ethnicity. The significant association of the “G” allele of SNP rs1048661 with PEG in the Saudi Arab population mirrors the rate observed in the Icelandic, Swedish, American, Australian, Austrian, German, Italian, Finnish, Japanese, and South African populations and differ from that observed in the Indian [14] and Chinese [15] populations (Table 5). In other studies, the opposite “T” allele of SNP rs1048661 was shown to be the risk allele for PEG in the Chinese [18] and Japanese [19] populations and thus raising doubts about the pathogenic role of SNP rs1048661.

**Table 5:** Summary of the genetics association of the two coding SNPs in the LOXL1 gene as reported in various populations.

** **	rs1048661 **“G” Allele**			rs3825942 **“G” allele**			** **
**Studied population**	**Case**	**Control**	**Significant association**	**Case**	**Control**	**Significant association**	**Reference**
American	0.819	0.600	0.000036	0.986	0.880	0.0003	[8]
American	0.787	0.665	0.0222	0.939	0.844	0.0194	[24]
American	0.843	0.703	7.74×10^−19^	0.959	0.798	3.10×10^−17^	[25]
American	0.829	0.719	0.0031	0.988	0.795	1.3×10^−13^	[26]
Australian	0.780	0.660	8.49×10^−4^	0.950	0.840	7.83×10^−5^	[9]
Austrian	0.841	0.671	<0.001	0.994	0.817	<0.001	[10]
Chinese	0.542	0.444	0.142	0.992	0.918	0.0018	[15]
Finnish	0.825	0.683	2.65×10^−5^	0.968	0.823	2.24×10^−8^	[27]
Germany	0.818	0.644	3.16×10^−8^	0.951	0.857	6.5×10^−13^	[11]
Iceland	0.781	0.651	1.8×10^−6^	0.984	0.847	4.1×10^−9^	[6]
Indian	0.721	0.634	0.156	0.923	0.742	0.0001	[14]
Italian	0.825	0.693	2.90×10^−19^	1.000	0.821	1.28×10^−40^	[12]
Japanese	0.005	0.554	>0.05	0.993	0.806	1.7×10^−8^	[19]
South African	0.990	0.810	1.7×10^−5^	0.130	0.620	5.2×10^-13•^	[23]
Swedish	0.834	0.682	2.7×10^−7^	0.995	0.879	9.1×10^−14^	[6]
Saudi Arabian	0.876	0.762	0.0056	0.968	0.817	0.000005	This study

^•^The p value reported here is in favor of the controls as the G allele was more frequent in controls (0.620) than in patients (0.130). In the South African population, the opposite allele “A” was the risk allele [23].

This study confirms a significant association between the “G” allele of SNP rs3825942 in cases versus controls (p=0.000005). Despite our in silico analysis using PolyPhen which predicted that this SNP was possibly damaging and that the amino acid glycine was highly conserved at codon 153 in many species, some doubts about the pathological role of this SNP in PEG were raised. This stems from previous reports that this SNP does not appear to affect *LOXL1* gene expression levels in blood or ocular tissues [6,22]. Additionally, the recent study in a South African population [16], where the “G” allele of SNP rs3825942 was protective and the opposite allele “A” was the risk allele for PEG, raises further uncertainties about the pathological role of this SNP.

Sequencing the coding region of the *LOXL1* gene revealed two previously unreported SNPs (g.25722 C>G and g.28084 T>G) detected in PEG patients and not in the controls. In silico analysis using PolyPhen predicted that g.25722 C>G is benign and that g.28084 T>G was probably damaging. We cannot be certain what role these two non- synonymous SNPs play in the development of PEG, although it is possible that either one of these two SNPs can be used for PEG risk assessment.

In summary we showed that the “G” allele of *LOXL1* SNPs rs1048661 and rs3825942 are associated with PEG in the Saudi populations in a fashion similar to other non-African populations.
